# Temporary mesenteric venous shunting for portal vein reconstruction: A novel technical adjunct

**DOI:** 10.1016/j.jvscit.2024.101540

**Published:** 2024-05-18

**Authors:** Jon G. Quatromoni, Robert Roses, Major K. Lee, Oksana A. Jackson, Benjamin M. Jackson, Ann C. Gaffey

**Affiliations:** a Department of Vascular Surgery, The Cleveland Clinic Foundation, Cleveland, OH; b Division of Endocrine and Oncologic Surgery, University of Pennsylvania Health System, Philadelphia, PA; c Division of Gastrointestinal Surgery, University of Pennsylvania Health System, Philadelphia, PA; d Division of Plastic Surgery, University of Pennsylvania Health System, Philadelphia, PA; e Division of Vascular and Endovascular Therapy, Lehigh Valley Heart and Vascular Institute, Allentown, PA; f Division of Vascular and Endovascular Surgery, Department of Surgery, University of California-San Diego, La Jolla, CA

**Keywords:** Intraoperative venous shunting, Portal vein reconstruction, Oncological reconstruction

## Abstract

Pancreatic resection not infrequently requires portal vein (PV) repair or replacement. PV reconstruction often requires bypass grafting or patch venoplasty, and these grafts and patches require time to thaw or harvest. Mesenteric ischemia and congestion with associated bowel edema may result from prolonged venous occlusion during thawing, harvesting, and reconstructing. Temporary shunting of the mesenteric venous circulation may mitigate these adverse effects. Twenty-one patients were shunted using Argyle shunts during PV reconstruction from 2010 to 2020. Reconstructions in this series consisted of aortic homograft interposition grafts (52%), bovine pericardial patches (38%), internal jugular vein interposition grafts (5%), and internal jugular patches (5%). No intraoperative complications resulted from shunt placement; technical success of PV reconstruction was 100%. Temporary venous shunting during PV reconstruction is safe, technically straightforward, and may serve to decrease the duration of venous mesenteric occlusion.

Cancers of the pancreas and hepatobiliary tree carry a poor prognosis overall.^1^ Complete surgical resection is the only curative treatment option.^2^^,^^3^ Significant advances in chemoradiation therapy have increased the pool of patients eligible for surgical resection.^3^^,^^4^ Driven by local regression and downstaging of previously inoperable, locally advanced disease, this population is characterized by an increased proportion of portal venous (PV) involvement.^5^ Hence, vascular surgeons are often called upon to perform mesenteric venous reconstruction.^3^^,^^6^^,^^7^

Once associated with poor outcomes and survival,^8^ PV reconstruction now plays an important role in achieving an R0 resection.^9^, ^10^, ^11^ Mesenteric venous reconstruction, although often necessary for a complete resection, takes time to perform. During this period, mesenteric ischemia is ongoing owing to the need to clamp the mesenteric veins. Prolonged venous occlusion can occur in cases requiring more complex reconstruction, particularly if a bypass graft must be thawed (cadaveric homograft) or harvested (femoral vein or internal jugular [IJ] vein). During the time for preparation and reconstruction, the bowel is subject to venous congestion and sequalae of such ischemia.

Shunting, a technique well-known to vascular surgeons from carotid endarterectomies and traumatic vessel injuries,^12^^,^^13^ can be applied to the mesenteric system during reconstruction. The main principle is to permit blood flow around the occluded segment under reconstruction, thereby continuing normal perfusion and reducing end organ edema and hypoperfusion. In the context of PV reconstruction, mesenteric ischemia is the byproduct of the interruption of mesenteric venous flow. No literature to date has reported the application of shunting in this new context. Thus, the goal of this study was to review a single institution's experience with the novel application of shunting during complex mesenteric venous reconstruction. We hypothesized that temporary mesenteric venous shunting during portal vein reconstruction (PVR) could be performed safely and with minimal complications.

## Methods

With approval from the Institutional Review Board of the Hospital of the University of Pennsylvania, a retrospective chart review was performed from a prospectively maintained database. All patients who underwent temporary procedural shunting of the mesenteric venous circulation during mesenteric venous reconstruction between 2010 and 2020 were examined. Demographic and clinical data were reviewed. Outcome measures included intraoperative complications resulting from shunt placement and intraoperative technical success of shunting and of PV reconstruction (defined as no intraoperative death and patent PVR assessed by intraoperative Doppler examination).

### Surgical technique

Before oncologic resection of the specimen, the degree of PV involvement is assessed intraoperatively alongside the hepatobiliary surgical team and—when possible—a joint decision made about the required repair. The tissue used for patch or graft conduit is then harvested if autologous, or prepared (for a frozen homograft, this requires 30 minutes for thawing and preparation). However, in the urgent or emergent setting owing to unexpected bleeding during the dissection, mesenteric venous clamps are applied before thawing or harvest. The emergent need for shunting accord in three patients. The decision for intraoperative anticoagulation before clamping depends on the extent of prior intraoperative blood loss and is left to the judgment of the individual surgeon.

For segmental resection of the PV and superior mesenteric vein (SMV), the proximal and distal ends of the resected vein are sent for frozen pathological examination, but reconstruction is not delayed during this analysis. The graft conduit is marked to maintain its orientation and prevent twisting, trimmed to the appropriate length, and passed over a #12 or #14 Argyle shunt. The shunt is inserted into the PV cranially and allowed to back bleed before securing it with a Rommel tourniquet. The shunt is placed into the PV first; we found that it was a bit more challenging to place given the fixed nature of the liver and often the short segment of PV that is left. The shunt is then temporarily clamped. The other end of the shunt is then inserted into SMV caudally and secured in place with an additional Rommel tourniquet before being unclamped to restore mesenteric venous flow. Flow is confirmed with a handheld audible Doppler.

The PV anastomosis is then performed first in an end-to-end fashion with running Prolene suture followed by the SMV anastomosis in a similar manner (Fig). The PV anastomosis is performed first, because we want to ensure circumferential 360° mobility of the graft to ensure that any backwall bleeding is identified—the PV anastomosis is the more challenging one. In our experience, visualization and assessment of the PV anastomosis after its completion was not limited significantly by the indwelling shunt. Furthermore, the degrees of freedom to evaluate the PV anastomosis offered by this approach (with the inherent laxity in the graft and shunt) is still greater when compared with the alternative scenario in which the graft has already been cut to length, pressurized, and fixed in its anatomical final resting position.

**Fig:**
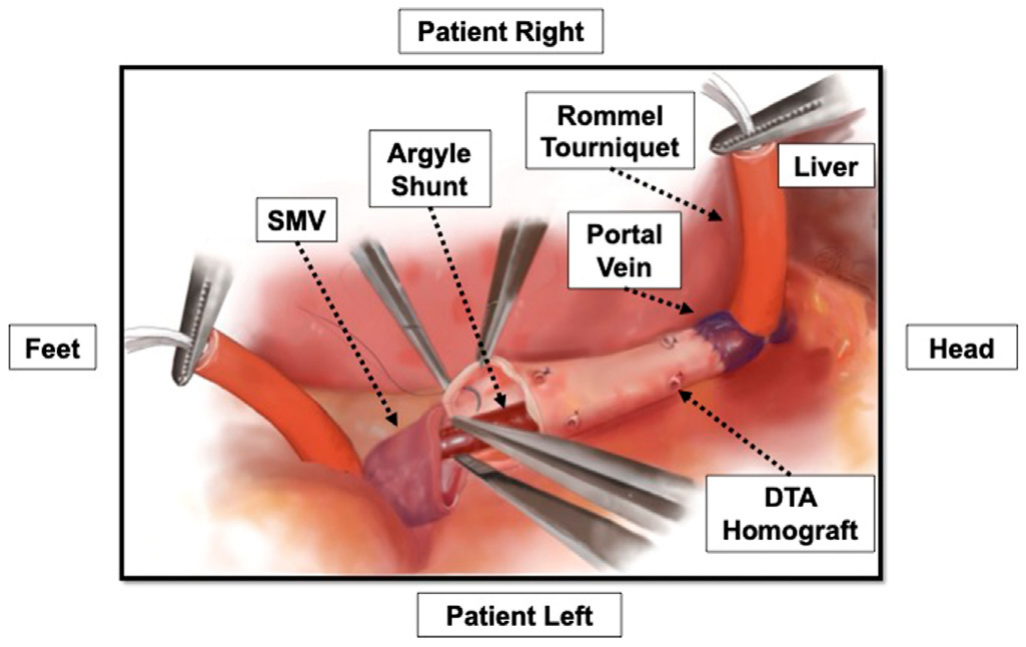
When we shunt for interposition grafts, the graft is placed over a #14 Argyle shunt, and then the shunt is inserted into the portal vein (*PV*) cranially and the superior mesenteric vein (*SMV*) caudally before being unclamped to restore mesenteric venous flow. We then complete the PV anastomosis first followed by the SMV anastomosis. Before the conclusion of the latter suture line, the shunt is removed.

Before conclusion of the latter suture line, the shunt is removed and the conduit clamped cranially and the SMV caudally to allow completion of the anastomosis. An audible Doppler is used to confirm adequate PV flow; assessing the pulsatility of the flow is aided by holding ventilations during Doppler interrogation. In addition, distention of the caudal (peripheral or intestinal) mesenteric veins is assessed visually and by palpation to ensure there is no venous hypertension upstream.

For partial PV resection amenable to patch angioplasty repair, the shunting sequence proceeds in a similar manner. The patch is then sewn in place using running Prolene sutures, and the shunt is removed before the completion of the suture line.

When possible, the splenic vein is preserved in continuity with the PV, if the tumor primarily involves the SMV. If the splenic vein-SMV confluence must be sacrificed, the splenic vein can sometimes be reimplanted onto the SMV-PV reconstruction. Frequently, however, the splenic vein is sacrificed.

## Results

Twenty-one patients, 12 females and 9 males, underwent temporary mesenteric venous shunting during oncologic resection and PV reconstruction from 2010 to 2020. Average age was 64 years. All patients had either pancreatic cancer (95%) or cholangiocarcinoma (5%). The majority of patients underwent neoadjuvant chemotherapy or radiation therapy (67%); 38% of the cohort had adjuvant therapy. Table I summarizes the complete demographic information.

**Table I:** Patient characteristics

	No. (%) or mean (min-max)
Total patients	21
Age	64 (46-82)
Female sex	11 (57)
Oncologic diagnosis	20 (95)
Pancreatic cancer	1 (5)
Cholangiocarcinoma	
Chemotherapy or radiation therapy	14 (67)
Neoadjuvant	8 (38)
Adjuvant	

The majority of patients underwent a Whipple procedure (81%); the remaining minority underwent total pancreatectomy and splenectomy (19%). Reconstructions consisted of interposition grafting (52% cadaveric descending thoracic aortic homograft and 5% IJ vein) or patch angioplasty (38% bovine pericardium and 5% IJ vein). Patients were heparinized systemically before PV clamping in 62% of cases (n = 13), locally heparinized in 19% (n = 4), and not anticoagulated in 19% of cases (n = 4). Either #12 and #14 Argyle shunts were used, depending on the caliber of the mesenteric vein being cannulated. Vascular surgical intervention was considered urgent or emergent in three of the cases (14%) owing to significant bleeding during the initial dissection. The splenic vein was ligated in 33% of cases (n = 7). For the six patients for whom data were available, the average mesenteric venous clamp time was 66 minutes (range, 36-130 minutes). Table II summarizes the procedural details for the study population.

**Table II:** Procedural details

	No. (%)
Index operation	17 (81)
Whipple procedure	4 (19)
Pancreatectomy and splenectomy	
PV reconstruction technique	9 (43)
Patch angioplasty	8 (38)
Bovine pericardium	1 (5)
IJ vein	12 (57)
Interposition graft	11 (52)
Cadaveric homograft	1 (5)
Internal jugular vein	
Argyle shunts (#12 or #14)	100%
Urgent/emergent status	3 (14)

*IJ*, Internal jugular; *PV*, portal vein.

In all but two patients, the shunt was placed easily into the SMV caudally and the PV cranially. One patient was shunted caudally into the splenic vein, because his SMV had chronic nonocclusive thrombus precluding shunt placement; in this case, the aim of the reconstruction was to maintain patency of splenic vein-to-PV flow. The other patient in whom shunting failed lacked sufficient length of the SMV stump to clamp around the shunt. No intraoperative complications resulted from shunt placement; there were no cases of vessel injury, perforation, thrombosis, or embolization. Intraoperative PV reconstruction technical success was 100%. Two patients, however, did require PV reclamping after the initial reconstruction was complete for distinct reasons unrelated to shunt placement. The first patient required conversion to an interposition descending thoracic aortic homograft from patch angioplasty after residual tumor was noted to be present at the PV margin. The second patient required a local thrombectomy in the setting of a prolonged PV clamp time despite prior local heparization (which had been clamped before initiation of reconstruction for hemostasis).

The postoperative course was uncomplicated for the patient with no returns to the operating room. The average time to return of bowel function was postoperative day 3 with the average discharge date of postoperative day 6. Furthermore, all patients were started on 81 mg aspirin when deemed to be safe from the surgical oncology team. All patients were also discharged on prophylactic lovenox for a total of 3 months given the high risk of thromboembolic events and in accordance with oncological guidelines.

## Discussion

Adenocarcinoma of the pancreas is the third leading cause of cancer death in the United States, with nearly 50,000 people dying of pancreatic cancer in 2019.^14^ Given that many of the pancreatic tumors are locally advanced at time of presentation, more aggressive surgical resections including PV reconstructions have been adopted. Diagnosis of PV/SMV involvement is mainly based on computed tomography imaging. Despite progress in imaging techniques, the nature of radiological involvement remains difficult to determine as perivascular inflammation may have the appearance of true vascular invasion on imaging. As such, despite the best preoperative planning, PV reconstruction may be unplanned, and bleeding may be encountered during pancreatectomy, resulting in prolonged and unanticipated PV occlusion for conduit harvest or preparation.

The most widely used method of vascular clamping within the portohepatic triangle is that described by Pringle in 1908, in which the hepatoduodenal ligament is clamped en masse.^15^, ^16^, ^17^, ^18^, ^19^ Although PV reconstruction typically only involves clamping of the mesenteric veins and not arterial occlusion, which is included in the Pringle maneuver, similar hemodynamic changes can be manifested. In particular, there is a linear increase in the risk of bowl ischemia owing to prolonged clamping of the mesenteric vessels with an associated reflex arterial constriction and occlusion. The concept of using an intraoperative arterial shunt to prevent prolonged ischemia is well-established in carotid endarterectomy and vascular surgery for traumatic injuries.^20^^,^^21^ In the former setting, vascular shunts can be applied based on objective measures of cerebral ischemia after clamping. In the latter setting, temporary vascular shunts can be useful for the prevention of tissue necrosis, compartment syndrome, and nerve injury until definitive arterial reconstruction is performed. In the setting of complex PV reconstruction—even when anticipated or planned such that conduit harvest or thawing can occur before mesenteric venous clamping—the reconstruction often takes 30 minutes or more, in which case shunting will presumably minimize intestinal ischemia, washout of toxins on unclamping, and bowel edema. In addition, mesenteric venous clamping, and the need for PV reconstruction, frequently cannot be predicted from preoperative imaging, and so conduit thawing, or harvest, cannot be accomplished before exposure and control of the venous circulation. Not infrequently, dissection and mobilization of tumors invading the PV, splenic vein, or SMV can result in injury and venous bleeding, at which point clamping the mesenteric veins often is necessary, and only at that junction does it become evident that venous reconstruction will be necessary, making shunting compelling during vein harvest or thawing of the homograft.

This study presents a new and straightforward method of venous shunting during complex oncological resection to decrease the systemic impact of PV occlusion. Interval shunting of the portal mesenteric system during PVR ensures physiologic portal flow throughout the operation. We observed no untoward events as a result of shunt placement. Especially during complex mesenteric venous reconstruction (eg, interposition bypass or patch angioplasty) in either a planned or emergent situation permits. Shunting ensures the avoidance of intestinal ischemia and edema is of the highest priority and the use of a temporary shunt is recommended to shorten the ischemic time and the deleterious impact of PV clamping.

A serious limitation of this series is the lack of a comparison control group consisting of a nonshunted cohort. However, this strategy was neither feasible nor informative, because the decision to shunt was made on a case-by-case basis according to the anatomical characteristics, the extent of oncologic resection, and the expected type and extent of PV reconstruction required. Specifically, at Penn, temporary shunting is used more commonly in more complex and prolonged mesenteric vein reconstructions. Also, it is difficult to identify clinically relevant end points to compare the two cohorts. Possible candidate variables included subjective assessment of bowel edema, liver function tests, lactate levels, return of bowel function, and ileus, but the variability in these measures is usually more reflective of events surrounding the oncologic portion of the procedure rather than of the shunt placement itself.

## Conclusions

Temporary vascular shunting is a well-established technique with which most vascular surgeons are comfortable and practiced. Applying it as an adjunct in a novel context for PV reconstruction is safe, technically straightforward, and may mitigate the deleterious effects of temporary mesenteric venous occlusion required for venous reconstruction during pancreatic resection.

## Disclosures

None.
